# A Common Control Group - Optimising the Experiment Design to Maximise Sensitivity

**DOI:** 10.1371/journal.pone.0114872

**Published:** 2014-12-11

**Authors:** Simon Bate, Natasha A. Karp

**Affiliations:** 1 Statistical Science Europe, GlaxoSmithKline Pharmaceuticals, Stevenage, United Kingdom; 2 Mouse Informatics Group, Wellcome Trust Sanger Institute, Cambridge, United Kingdom; National Institute of Environmental and Health Sciences, United States of America

## Abstract

Methods for choosing an appropriate sample size in animal experiments have received much attention in the statistical and biological literature. Due to ethical constraints the number of animals used is always reduced where possible. However, as the number of animals decreases so the risk of obtaining inconclusive results increases. By using a more efficient experimental design we can, for a given number of animals, reduce this risk. In this paper two popular cases are considered, where planned comparisons are made to compare treatments back to control and when researchers plan to make all pairwise comparisons. By using theoretical and empirical techniques we show that for studies where all pairwise comparisons are made the traditional balanced design, as suggested in the literature, maximises sensitivity. For studies that involve planned comparisons of the treatment groups back to the control group, which are inherently more sensitive due to the reduced multiple testing burden, the sensitivity is maximised by increasing the number of animals in the control group while decreasing the number in the treated groups.

## Introduction

The 3R's, Replacement, Reduction and Refinement, introduced as a framework for achieving the most humane treatment of experimental animals, has been widely accepted as a prerequisite for a successful animal experiment [1]. Attention on the refinement element of the framework has been growing in recent years. Refinement refers to improvements to scientific procedures and husbandry which minimise actual or potential pain, suffering, distress or lasting harm and/or improve animal welfare in situations where the use of animals is unavoidable. In 2009, Kilkenny *et al*. published a systematic review of published papers involving *in vivo* experiments and highlighted that many published experiments did not use the most appropriate experimental design. For example, in the survey only 62% of experiments that should have employed a factorial design had in fact done so [2]. Experimental design and statistical analysis fall under the refinement element of the 3R's as they reduce further experimentation and ensure that the animals used fulfil the goals of the experiment. This has led to the publication of the Animal Research: Reporting *In Vivo* Experiments (ARRIVE) guidelines [3], a checklist that aims to embed good practice in the experimentation process. The impact of poor experimental design can be profound, as shown by a systematic study that found a lack of concordance between animal experiments and clinical trials [4]. The authors concluded that majority of the animal studies were of poor methodological quality. In practice though poor design and analysis is not restricted to animal experimentation and is thought to be endemic throughout scientific research [5], [6].

In science and statistics, validity is the extent to which a conclusion or measurement is reliable and corresponds accurately to the real world. The validity of an experiment can be evaluated in many ways. For example, the conclusion validity is the degree to which conclusions we reach about our data are reasonable [7] and relates to the experiment's ability to assess the relationship. The majority of studies involving animals use statistical hypothesis testing, where a *p*-value is calculated to assess whether the null hypothesis (of no effect) can be rejected and hence the alternative hypothesis (the effect you are trying to prove) accepted. With the use of inferential hypothesis testing, there is potential to conclude there is an effect when in fact there is none – a false positive (type I error). Conversely, there is potential to conclude there is not an effect when in fact there is one (type II error). When considering the type II error rate it is often more useful to consider the statistical power (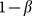, where 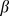 is the probability of a type II error occurring). The statistical power is the probability (or chance) of achieving a statistically significant result when conducting a study given that, in reality, there is a real effect [8]. In practice a power in excess of 80% is usually considered acceptable. With experiments involving animals it is critical to ensure that the experiment has sufficient power so that not only real effects are detected, but also that the experiment is not over-resourced such that animals are wasted [9].

Frequently, animal researchers conduct experiments that involve multiple treatments and a common control. For example, a survey of recent PLoS ONE papers identified an R&D drug study involving multiple different treatments versus a vehicle control [10], a study comparing high cholesterol diets to a low cholesterol diet [11] and a study comparing responses at later time points to a baseline group [12]. This type of study design is also commonly used in toxicology and safety assessment where studies are typically performed so that they can compare increasing doses of a treatment back to a control group. For example, Lee *et al*. [13] describe a repeated oral dose toxicity study in rats to compare three doses of KMS88009 back to a vehicle control. In these experiments comparisons back to the control will be the only comparisons that are of interest, regardless of the experimental results. It is important to note that the researcher plans which comparisons that wish to make in advance – they are examples of so-called planned comparisons [14], as opposed to general ‘post hoc testing’ which involves making all pairwise comparisons). Planned comparisons are beneficial for two reasons. Firstly, the decision regarding which tests to perform is made before the data is collected and hence is not influenced by the observed results. In theory, this should reduce the risk of inadvertently finding false positive results in a ‘data-trawling’ exercise. Secondly, planned comparisons increase the sensitivity of the experiment as it reduces the multiple testing burden. The multiple testing burden arises because the chance of finding a false positive, for a given significance threshold, accumulates with each statistical test conducted. If all pairwise comparisons are performed, for example using an LSD (Least Significant difference) test [8], then there is an increased risk of finding false positives. To manage this risk a more stringent threshold is applied; by making a multiple comparison adjustment to the LSD p-values. Consider the scenario with one control group and three treatments. If all groups are compared then the post-hoc testing would involve six separate pairwise statistical comparisons. However, if planned comparisons of treatments back to control are performed then this corresponds to only three separate statistical comparisons and the threshold adjustment would be less. In this paper, we shall consider the implications on the choice of design when the researcher knows in advance which comparisons they wish to make.

When constructing experimental designs that involve a number of treatment groups and a control group, interest rightly focuses on the sample size that is required in each of the experimental (treatment and control) groups. It appears to be standard practice to assign the same number of animals to each of the experimental groups (the so-called ‘balanced’ designs). Such practice is perhaps encouraged by sample size calculation software, where typically one sample size is recommended across all groups [15], [16]. The statistical test applied also influences the sample size required. A common approach used to analyse data generated from these experiments, assuming the parametric assumptions hold, is to compare the treatment group means to the control group mean, using either *t*-tests, Analysis of Variance followed by Dunnett's test or applying a multiple comparison adjustment to the LSD p-values. It is therefore common practice to perform a sample size calculation under the assumption that the statistical analysis will be performed using one of these tests [17].

In this paper, we shall use optimal design theory to investigate the effects of varying the replication of the experimental groups. We shall assume that the data will be analysed using either multiple *t*-tests or Analysis of Variance followed by a suitable multiple comparison procedure. Crucially we differentiate between the experimental situations where the researcher only plans to compare the treatments back to control and when they plan to make all pairwise comparisons. We will focus on the former case, and highlight how different experimental designs result in different levels of statistical power.

## Methods

Two approaches are considered in this paper to investigate the effect of varying the control group replication; a theoretical investigation and a power comparison.

### Theoretical approach to maximising sensitivity

For the theoretical investigation, we need to make a few assumptions. While restrictive, many animal experiments satisfy these assumptions. We assume that:

1. The researcher conducts an experiment to either (a) compare 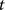 treatments to a single control or (b) make all possible pairwise comparisons between the experimental groups. The experimental design therefore involves 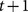 experimental groups.
2. A total of 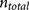 animals are used in the experiment and they are allocated at random to the 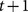 experimental groups.
3. The replication in the 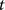 treatment groups is the same 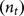.
4. The replication in the control group is 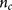.
5. The variability of the responses is the same across all experimental groups. In practice the response may require a transformation in order to satisfy this condition.
6. The parametric assumptions hold (for example, the responses are numerical, independent, continuous and the residuals are normally distributed) and hence a parametric test, such as the *t*-test or Analysis of Variance followed by pairwise comparisons, will be used to compare the experimental groups.

By considering the predicted standard error of the estimates of the comparisons of interest, when using a given experimental design, it is possible to compare and contrast different designs. The more efficient the design, the smaller the predicted standard errors will be and hence the statistical tests will be more sensitive. For a given total number of animals, we use mathematical arguments (see S1 Derivations for more details) to investigate how varying the replication of the control group influences these standard errors.

### Power analysis assessment

To highlight the practical implications of using different experimental designs, we investigate the statistical power that can be achieved when comparing all treatments back to a single common control using planned comparisons. The tests within this manuscript are not adjusted for multiplicity, as the adjustment needed varies between the analysis scenarios and adds complexity to the analysis. The absolute level of statistical power is not of direct interest; rather we are interested in investigating how varying the experimental design (control group replication) influences its statistical power.

For a given level of variability 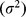, a difference between the two group means 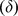, a significance level of 5% and sample sizes 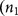 and 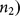, the power 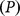 of a two-sided test that is not adjusted for multiplicity is given by
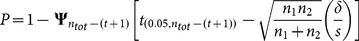
(1)where 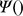 is the cumulative density function (CDF) of the t distribution with 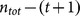 degrees of freedom and 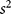 is an estimate of the variance [14]. The derivation of this formula is given in S2 Derivations.

Using (1) we investigate the power that can be achieved in various real-life situations. For convenience the total number of animals included in each situation is selected so that 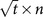 (where 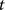 is the number of treatment groups and 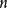 is the replication in the treatment groups) is approximately an integer.

## Results

### Theoretical approach to maximising sensitivity

Using the mathematical arguments given in S1 Derivations we can, for a variety of scenarios, assess the optimal replication in the control group to achieve for maximum sensitivity. We assume that the researcher is running an experiment that satisfies the five conditions discussed in the methods.

#### Scenario 1

Assume that the only comparisons the researcher plans to make involve comparing the treatment groups back to the control group. For a given total number of animals 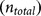, if there are 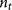 animals in each of the 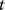 treatment groups and 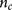 animals in the control group, then let there be 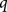 more animals in the control group compared to the treated groups, i.e.
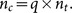
and
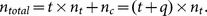



Note if 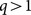 then there are more animals in the control group compared to the treated groups and if 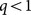 then there are fewer animals in the control group compared to the treated groups.

The estimates of the pairwise comparisons of interest are as precise as possible if:
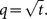



In other words, the number of animals in the control group should be 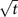 times the number in the treatment groups. So in an experiment involving comparing four treatment groups back to a control group, then twice as many animals should be allocated to the control group than are allocated to the treatment groups.

#### Scenario 2

Assume that the researcher is interested in making all possible pairwise comparisons between the 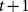 experimental groups. It turns out that these comparisons are estimated as precisely as possible if: 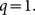



In other words, as expected by symmetry, as all groups are involved in the same number of comparisons, the same number of animals should be allocated to each of the experimental groups (treatment groups and the control group).

From consideration of these two scenarios, we can see the optimal design depends on the goal of the experiment. With the defined planned comparisons in Scenario 1, an unbalanced design with more animals allocated to the control group results in comparisons that are estimated more precisely. This gain in sensitivity is at the expense of treatment comparisons that the researcher does not plan to make. In other words, the pairwise comparisons of interest are more precise, everything else being equal, if one design is employed when compared to another. From a less mathematical point of view, these results make sense as the control group is used more often than the other treatment means, and hence it is important to have a precise estimate of the control group mean.

### Power analysis assessment for Scenario 1

We shall now consider Scenario 1 in more detail. The previous analysis identified the optimal design to maximise sensitivity and we now focus on quantifying the impact on the statistical power of the various designs.

**Figure 1:**
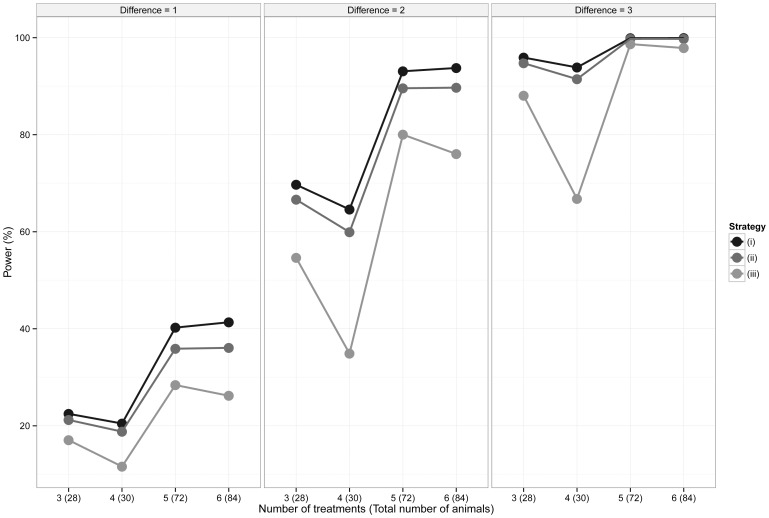
Statistical power of various levels of replication of the control group as the biological effect increases. The variability of the responses is fixed at 2.25. Three strategies for selecting the size of the control group were considered: (i) Optimal, according to the theoretical derivation, (ii) Equal to the treatment groups and (iii) Less than, where the control group replication is less than the treatment groups.

**Figure 2:**
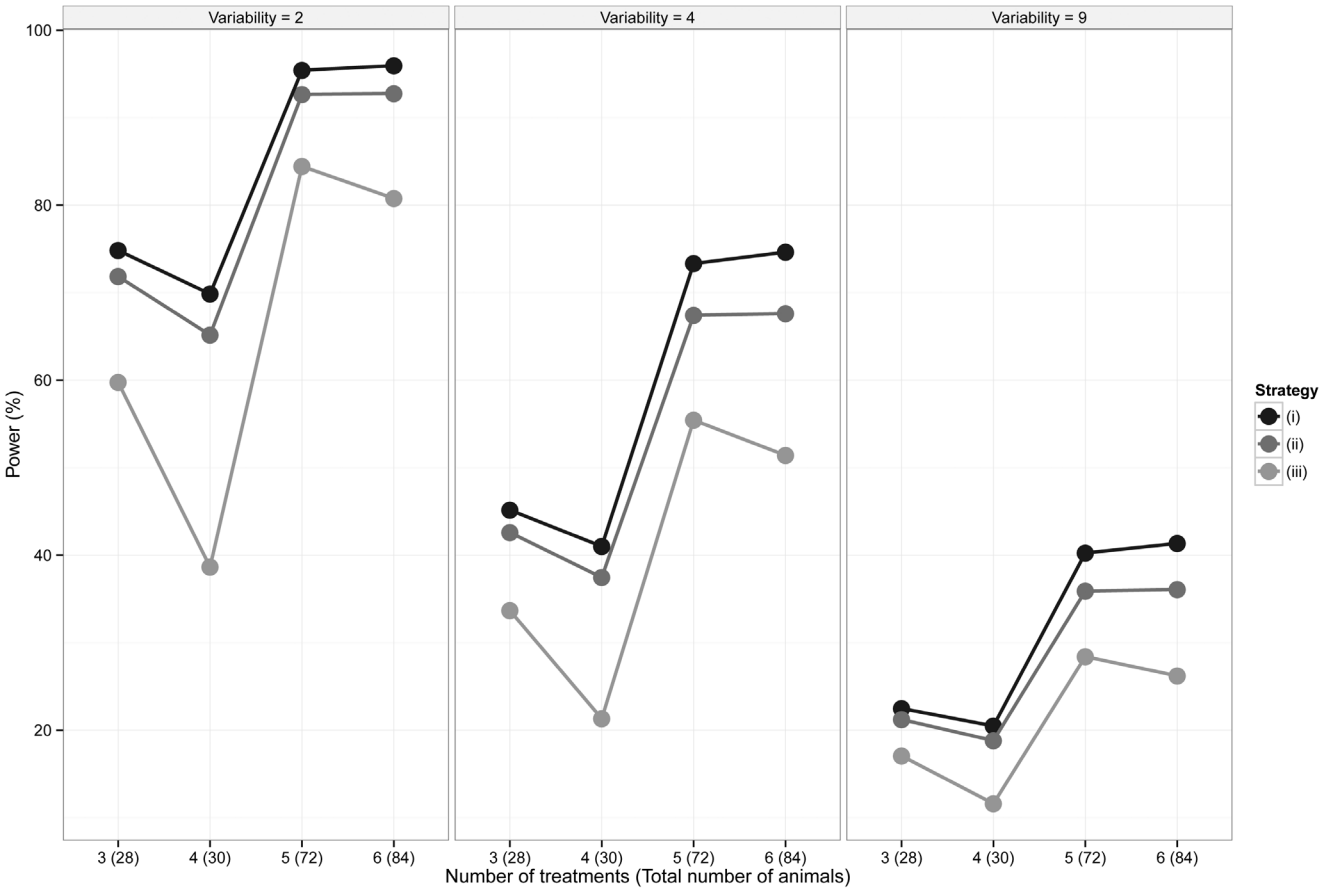
Statistical power of various levels of replication of the control group as the variability increases. The difference between the treatment and control groups is fixed at 2. Three strategies for selecting the size of the control group were considered: (i) Optimal, according to the theoretical derivation, (ii) Equal to the treatment groups and (iii) Less than, where the control group replication is less than the treatment groups.

Using equation (1), the statistical power of various levels of replication of the control group was investigated by assessing various designs when the size of the biological effect increases for a defined amount of biological variability (Table 1 and Fig. 1) and when the size of the biological effect is fixed but the biological variation increases (Table 2 and Fig. 2). Three control group replication strategies are considered: when the replication in the control group is (i) 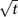 more than the treated groups - the theoretically optimal solution, (ii) equal to the replication in the treated groups and (iii) less than the replication in the treated groups. While (i) has been shown to be the optimal solution, (ii) and (iii) are commonly applied in practice and hence it is of interest to consider how these designs compare to the theoretically optimal design. To give context, the biological effect being tested for each calculation has been presented as a standardised effect size (Cohen's *d* or *Z* statistic) where the biological effect is scaled relative to the biological variability (i.e. 1 equals a differences equivalent to one unit of variability) [18].

**Table 1:** Statistical power of various levels of replication of the control group as the biological effect increases.

Number of treatment groups	Control Group Replication Strategy†	Treatment group replication	Control group replication	Total number of animals	Difference between the treatment and control groups (Absolute size, Cohen's *d*)		
		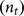	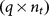	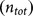	(1, 0.67)	(2, 1.33)	(3, 2)
3	(i)	6	10	28	22.47%	69.70%	95.91%
	(ii)	7	7	28	21.21%	66.63%	94.75%
	(iii)	8	4^a^	28	17.05%	54.64%	88.05%
4	(i)	5	10	30	20.47%	64.60%	93.87%
	(ii)	6	6	30	18.80%	59.92%	91.46%
	(iii)	7	2^b^	30	11.60%	34.89%	66.78%
5	(i)	10	22	72	40.24%	93.08%	99.91%
	(ii)	12	12	72	35.89%	89.58%	99.75%
	(iii)	13	7^c^	72	28.40%	80.03%	98.68%
6	(i)	10	24	84	41.34%	93.76%	99.93%
	(ii)	12	12	84	36.07%	89.70%	99.76%
	(iii)	13	6^d^	84	26.20%	76.03%	97.87%

The variability of the responses is fixed at 2.25. Three strategies for selecting the size of the control group were considered: (i) Optimal, according to the theoretical derivation, (ii) Equal to the treatment groups and (iii) Less than, where the control group replication is less than the treatment groups.

† : for control group replication strategy (i) 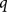 is approximately 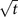, (ii) 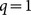 and (iii) 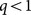,

specifically: a: 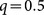, b: 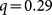, c: 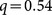 and d: 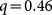.

**Table 2:** Statistical power of various levels of replication of the control group as the variance increases.

Number of treatment groups	Control Group Replication Strategy†	Treatment group replication	Control group replication	Total number of animals	Variability of the responses (Variance, Cohen's *d*)		
		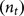	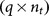	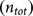	(2, 1.41)	(4,1)	(9, 0.67)
3	(i)	6	10	28	74.83%	45.15%	22.47%
	(ii)	7	7	28	71.85%	42.59%	21.21%
	(iii)	8	4^a^	28	59.76%	33.67%	17.05%
4	(i)	5	10	30	69.85%	41.00%	20.47%
	(ii)	6	6	30	65.16%	37.45%	18.80%
	(iii)	7	2^b^	30	38.64%	21.31%	11.60%
5	(i)	10	22	72	95.42%	73.33%	40.24%
	(ii)	12	12	72	92.66%	67.42%	35.89%
	(iii)	13	7^c^	72	84.44%	55.43%	28.40%
6	(i)	10	24	84	95.94%	74.63%	41.34%
	(ii)	12	12	84	92.76%	67.61%	36.07%
	(iii)	13	6^d^	84	80.77%	51.40%	26.20%

The difference between the treatment and control groups is fixed at 2. Three strategies for selecting the size of the control group were considered: (i) Optimal, according to the theoretical derivation, (ii) Equal to the treatment groups and (iii) Less than, where the control group replication is less than the treatment groups.

† : for control group replication strategy (i) 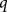 is approximately 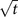, (ii) 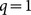 and (iii) 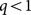,

specifically: a: 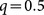, b: 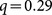, c: 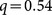 and d: 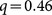.

From Tables 1 and 2 it can be seen that, in the situations considered, a gain in statistical power of between 0.16% and 7.02% (with a median of 3.12%) can be achieved when using the mathematically optimal replication of controls, compared to replicating all groups equally. This benefit is reduced if the statistical power obtained when using both designs approaches 100%. While such improvements are perhaps of marginal practical importance, especially in suitably powered experiments, it is still the case that a slight change to the experimental design can result in more sensitive statistical tests without increasing the total number of animals used.

Perhaps more strikingly, from Table 1 and 2 it can be seen that there is a significant drop in statistical power if the number of animals in the control group is less than the number in the treatment groups. For example, if a design is required to compare four treatments with a control, the size of biologically relevant effect is 2 and the variability of the responses is 2.25, then a 30% increase in power can be achieved if the optimal replication of animals is used, when compared to a design where there are fewer animals in the control group compared to the treated groups.

## Conclusions

A review of the literature seems to imply the researcher should use a balanced design with the same number of animals allocated to each experimental group. For example, Ruxton and Colegrave [19] state “Always aim to balance your experiment, unless you have a very good reason not to”. In many statistical texts the sample size calculation is performed when the experimental design consists of only two groups. In this case the balanced design is usually a sensible choice. Unfortunately designs used in practice are rarely so straightforward, and hence the orthodox strategy may not always be appropriate.

In this paper we have shown the benefit that can be gained from using an experimental design that has been constructed to favour the comparisons that the researcher plans to make. Focusing on the comparisons of interest increases sensitivity as it reduces the adjustment that is required to manage the multiple testing burden (i.e. reduce the false positive risk). In the case considered in this paper, where the researcher wishes to compare *t* treatments to a control, the design should be selected so that the number of animals in the control group is 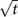 times the number in the treated groups. It has been shown that such a design performs better than the commonly used strategy of equally replicating across the treatment and control groups. While beyond the scope of this paper, a similar approach can be used for the more complicated experimental designs. For example, the choice of block or cross-over design can influence the reliability of the treatment comparisons.

Another strategy that researchers may follow when designing their experiments is to reduce the number of animals in the control group compared to the treatment groups. This approach is usually taken because the researcher has access to historical control data and feels that this knowledge implies fewer concurrent controls are needed. There has been much written about the benefit of using historical control data when assessing the effect of treatments [20] though it does not replace concurrent controls. Prior knowledge, perhaps obtained from a historical control database, can also be incorporated into the statistical analysis using a Bayesian analysis paradigm. This approach has been successfully applied in clinical research, although such studies usually involve comparing a single treatment to a control or placebo [21]. While there are certain benefits to comparing multiple treatments to a historical control group, this work highlights that a study with both concurrent and historic controls does not necessarily imply that fewer animals can be included in a concurrent control. When treatments are compared using the popular statistical analysis approaches discussed in the methods section, we have demonstrated that having more animals in the treatment groups, compared to the control group, can lead to a significant reduction in statistical power regardless of the benefits of using historical control information.

In this paper we have assumed that the variability is the same in all experimental groups. In practice this assumption may not hold. For example, in biological responses it is common for the variability to increase with the size of the response. Furthermore, responses that are bounded (e.g. percentages which cannot go below 0 or above 100) tend to be less variable as a boundary is approached. In such cases, there are statistical analysis strategies that can be applied, but they are beyond the scope of this paper. An alternative strategy, commonly recommended [8], [14], [22], is to investigate the use of non-linear data transformations to “correct” the data which then allow application of the methods discussed within this paper. For example, the arcsine transformation for percentage data, square root transformation for count data and log transformation if the variability increases as the response increases.

The arguments presented in this paper, also assume that any attrition due to experimental procedures is expected to be the same across all groups. If the researcher believes, for example, that they are likely to lose more animals in the treated groups, then they may wish to adjust the initial sample sizes so that the sample sizes achieved at the end of the study should result in a design that is close to the optimal design.

In practice, if the experiment is to be successful, many considerations should be taken into account when constructing a design. Issues such as practical constraints on the experimental material, financial pressures and ethical issues should be taken into account alongside optimal statistical design theory. In this paper we have aimed to highlight what a theoretically optimal experimental design, all things being equal, would be. The researcher should use this knowledge, in conjunction with other constraints, when planning their experiment.

## Supporting Information

S1 Derivations Determining the optimum control group **replication.**
(DOCX)

S2 Derivations Determining the statistical power.
(DOCX)
