# Supplementary material for: A Common Control Group - Optimising the Experiment Design to Maximise Sensitivity
Source: PLoS One. 2014 Dec 11;9(12):e114872. doi: 10.1371/journal.pone.0114872 (PMC4263717; doi:10.1371/journal.pone.0114872)
Supplement: S1 Derivations — Determining the optimum control group replication. (DOCX) [file pone.0114872.s001.docx]

# ****Derivations S1. Determining the optimum control group**** replication

**Theorem (Scenario 1)**

Consider an experiment involving of$t$treatment groups and a single common control group, where conditions 1-5 described in the results section of the paper hold. Assume that:

- The replication in all of the treatment groups$.$
- The replication in the control group$=n_{c}=q\times n_{t}$, where$q$is a positive number that is not necessarily an integer.
- The total number of animals in the experiment$n_{total}=t\times n_{t}+n_{c}=n_{t}(t+q)$*.*

We also assume that the purpose of the experiment is to compare the treatment group means back to control group mean (see Scenario 1) using a suitable parametric test [8].

The number of animals that should be allocated to the control group, so that the standard error of the estimate of the comparisons between each treatment group and the control group is minimised, is given by:

$$n_{c}=q\times n_{t}=\sqrt{t}\times n_{t}$$

i.e. $q=\sqrt{t}$

**Proof**

Consider the null hypothesis that there is no difference between the$i^{th}$treatment mean and the control group mean, i.e.

$$H_{0}:\tau_{i}-\tau_{c}=0$$

where$\tau_{i}$is the effect of the$i^{th}$treatment and$\tau_{c}$is the effect of the control.

Without loss of generality, we assess this hypothesis by considering comparison$\Delta_{1}$, where

$$\Delta_{1}=\overline{x_{i}}-\overline{x_{c}}$$

$\overline{x_{i}}$is the $i^{th}$ treatment mean and$\overline{x_{c}}$is the control group mean.

The standard error of this comparison$(SEM_{\Delta_{1}})$is given by

$$SEM_{\Delta_{1}}=\sigma\sqrt{\frac{1}{n_{t}}+\frac{1}{n_{c}}}=\sigma\sqrt{V}$$

where$\sigma^{2}$is the variance and

$$V=\frac{1}{n_{t}}+\frac{1}{n_{c}}=\frac{1}{n_{t}}+\frac{1}{q\times n_{t}}=\frac{1}{n_{t}}\left(1+\frac{1}{q}\right)$$

To obtain the most reliable estimate of$\Delta_{1}$, for a given level of variability and total sample size, we need to minimise$V$.

Now

$$n_{total}=n_{t}\times(t+q)$$

i.e.

$$n_{t}=\frac{n_{total}}{(t+q)}$$

Therefore, substituting (4) in (3) gives

$$V=\frac{1}{n_{t}}\left(1+\frac{1}{q}\right)=\left(\frac{t+q}{n_{total}}\right)\left(1+\frac{1}{q}\right)=\frac{t}{n_{total}}+\frac{q}{n_{total}}+\frac{t}{n_{total}\times q}+\frac{1}{n_{total}}$$

To find the value of$q$which minimises$V$we need to differentiate$V$with respect to$q$, and set to zero.

Now

$$\frac{dV}{dq}=\frac{1}{n_{total}}-\frac{t}{n_{total}\times q^{2}}$$

and$\frac{dV}{dq}=0$when

$$\frac{1}{n_{total}}-\frac{t}{n_{total}\times q^{2}}=0$$

i.e.

$q=\sqrt{t}$.

While this is a solution of$\frac{dV}{dq}=0$, it could be a minimum or maximum solution. To confirm it is the minimum solution, we differentiate (6) again, i.e.

$$\frac{d^{2}V}{dq^{2}}=\frac{2t}{n_{total}\times q^{3}}$$

As$q>0$,$t>0$and$n_{total}>0$this implies that

$$\frac{d^{2}V}{dq^{2}}>0$$

As$\frac{d^{2}V}{dq^{2}}>0$and$\frac{dV}{dq}=0$when$q=\sqrt{t}$we therefore know that$V$is minimised when$q=\sqrt{t}$.

**Theorem (Scenario 2)**

Consider an experiment involving of$t$treatment groups and a single common control group, where conditions 1-5 described in the results section of the paper hold. Assume that:

- The replication in all of the treatment groups$.$
- The replication in the control group$=n_{c}=q\times n_{t}$, where$q$is a positive number that is not necessarily an integer.
- The total number of animals in the experiment$n_{total}=t\times n_{t}+n_{c}=n_{t}(t+q)$*.*

If all pairwise comparisons are of interest to the researcher, i.e. not only the comparisons of treatments versus control but also the comparisons between the treatments themselves, then the replication in the control group should be

$$n_{c}=q\times n_{t}=n_{t}$$

i.e.$q=1$.

**Proof**

From equation (2) above the standard error of the estimate of a treatment versus control comparison$\Delta_{1}$is given by:

$$SEM_{\Delta_{1}}=\sigma\sqrt{\frac{1}{n_{t}}+\frac{1}{n_{c}}}$$

Similarly, the standard error of an estimate of a pairwise comparison of two treatments$(\Delta_{2})$is given by

$$SEM_{\Delta_{2}}=\sigma\sqrt{\frac{1}{n_{t}}+\frac{1}{n_{t}}}=\sigma\sqrt{\frac{2}{n_{t}}}$$

Now as all comparisons are of equal importance, we require the standard error of the estimates of all comparisons to be the same, i.e.$SEM_{\Delta_{1}}=SEM_{\Delta_{2}}$. Therefore

$$\sigma\sqrt{\frac{1}{n_{t}}+\frac{1}{n_{c}}}=\sigma\sqrt{\frac{2}{n_{t}}}$$

Cancelling-out$\sigma$in (9), squaring and replacing$n_{c}$by$q\times n_{t}$gives

$$\frac{1}{n_{t}}+\frac{1}{q\times n_{t}}=\frac{2}{n_{t}}$$

i.e.

$$1+\frac{1}{q}=2$$

which gives$q=1$.
