# Supplementary material for: A Common Control Group - Optimising the Experiment Design to Maximise Sensitivity
Source: PLoS One. 2014 Dec 11;9(12):e114872. doi: 10.1371/journal.pone.0114872 (PMC4263717; doi:10.1371/journal.pone.0114872)
Supplement: S2 Derivations — Determining the statistical power. (DOCX) [file pone.0114872.s002.docx]

**Derivations S2. Determining the statistical power**

Consider the situation where two independent samples are compared and define$\beta$such that$\beta=(1-P)$, where$P$is the statistical power. It can be shown that if the sample size is the same in each group$(n)$then

$$n=\left(t_{(\alpha/2,2n-2)}+t_{(\beta,2n-2)}\right)^{2}\left(2s^{2}/\Delta^{2}\right)$$

where$s^{2}$is an estimate of the variance,$\Delta$is the difference between the two samples,$t_{(\alpha/2,2n-2)}$is the two-tailed significance level of the$t$distribution at significance level$\alpha$with$2n-2$degrees of freedom [14].

Rearranging (10) gives

$$t_{(\beta,2n-2)}=\sqrt{\frac{n}{2}}\left(\frac{\Delta}{s}\right)-t_{(\alpha/2,2n-2)}$$

Now the standard error of the estimate of the difference between two samples$(\Delta)$of equal size$n$is given by

$$SEM_{\Delta}=s\sqrt{\frac{2}{n}}=s\sqrt{\frac{1}{n}+\frac{1}{n}}$$

Substituting (12) in (11) gives

$$t_{(\beta,2n-2)}=\frac{\Delta}{SEM_{\Delta}}-t_{(\alpha/2,2n-2)}$$

Now if the sample sizes are different,$n_{1}$and$n_{2}$say, then (12) can be generalised to

$$SEM_{\Delta}=s\sqrt{\frac{1}{n_{1}}+\frac{1}{n_{2}}}=s\sqrt{\frac{(n_{1}+n_{2})}{n_{1}n_{2}}}$$

Substituting (14) into (13), and replacing$2n$by$n_{1}+n_{2}$, gives

$$t_{(\beta,n_{1}+n_{2}-2)}=\sqrt{\frac{n_{1}n_{2}}{n_{1}+n_{2}}\left(\frac{\Delta}{s}\right)}-t_{(\alpha/2,n_{1}+n_{2}-2)}$$

Hence

$$P=1-\Psi_{n_{1}+n_{2}-2}\left[t_{(\alpha/2,n_{1}+n_{2}-2)}-\sqrt{\frac{n_{1}n_{2}}{n_{1}+n_{2}}}\left(\frac{\Delta}{s}\right)\right]$$

where$\Psi()$is the cumulative density function (CDF) of the$t$distribution with$n_{1}+n_{2}-2$degrees of freedom.
